# Predictive Factors for Access-Site Pain Chronicity after Percutaneous Coronary Intervention via Radial Artery Access

**DOI:** 10.1155/2020/8887499

**Published:** 2020-11-16

**Authors:** Liuda Brogiene, Giedre Baksyte, Agne Klimaite, Martynas Paliokas, Andrius Macas

**Affiliations:** ^1^Department of Anaesthesiology, Lithuanian University of Health Sciences, Kaunas, Lithuania; ^2^Department of Cardiology, Lithuanian University of Health Sciences, Kaunas, Lithuania

## Abstract

**Objectives:**

The aim of this study is to assess the prevalence and predictive factors for developing chronic access-site (A-S) pain after percutaneous coronary intervention (PCI) via radial artery access.

**Methods:**

Data of selected patients (*n* = 161) who underwent elective PCI were collected prospectively and analysed in 2020. Verbal analogue scale was used to evaluate pain intensity after 12, 24, and 48 h and 3 months after PCI. The univariate logistic regression analysis was used.

**Results:**

Pain prevalence decreased from 29% straight after PCI and 54% two hours later to 3.7% following 3 months after procedure. The predictors for A-S pain chronicity are diabetes (OR = 5.77 95% CI (1.07–31.08), *p* = 0.041), hematoma (OR = 6.48, 95% CI (1.06–39.66), *p* = 0.043), A-S hand neuropathy (OR = 19.93 95% CI (1.27–312.32), *p* = 0.033), A-S pain immediately after PCI (OR = 14.60 95% CI (1.63–130.27), *p* = 0.016), after 12 h (OR = 17.2 95% CI (1.60–185.27), *p* = 0.019), 24 h (OR = 48 95% CI (4.87–487), *p* = 0.01), and 48 h (OR = 23.46 95% CI (3.81–144.17), *p* = 0.001), and pain intensity immediately after procedure (OR = 3.30 95% CI (1.65–6.60), *p* = 0.001), after 2 h (OR = 2.56 95% CI (1.15–5.73), *p* = 0.022), after 12 h (OR = 3.02 95% CI (1.70–5.39), *p* < 0.001), after 24 h (OR = 3.58 95% CI (1.90–6.74), *p* < 0.001), and after 48 h (OR = 2.89 95% CI (1.72–4.87), *p* < 0.001). Pain control was performed with Ketoprofen and Ibuprofen as most used NSAIDs. 10 mg of Morphine intravenously was the choice from strong opioids if necessary.

**Conclusions:**

The prevalence of chronic A-S pain is 3.7%. Main predictive factors for the A-S pain chronicity are diabetes, hematoma, and persistent pain and pain intensity during 48 h period after PCI.

## 1. Introduction

Ischemic heart disease (IHD) is one of the leading life-threatening diseases in the world. Percutaneous Coronary Intervention (PCI) is the gold standard diagnostic and treatment providing tool for IHD. This procedure is performed mostly through the femoral or radial artery access. The transradial (TR) approach is commonly used because of its reduced life-threatening complications, risk, mortality, hospital stay, and better earlier mobilization after the procedure [1]. Despite good care, access-site (A-S) complications, such us bleeding, hematoma, artery spasm, pseudoaneurysm, or thrombosis accompanied by pain and possibly requiring urgent intervention, can occur [2–4].

It is known that postprocedural pain can occur after any procedure that causes actual or potential tissue damage. Individuals who undergo potentially painful procedures must have optimal pain management before, during, and after the procedure [5]. Recently, numbers of clinical cases, describing precisely debilitating pain syndromes after PCI via TR approach, are growing [6, 7]. There are studies describing prevalence of acute A-S pain after PCI via radial artery. Acute postprocedural arm pain occurs approximately in 1 out of 20 patients after PCI via TR approach [8]. Several studies reported severe periprocedural pain with prevalence of 4–10% [9–14]. The development of chronic A-S pain after PCI is not described in the literature, but it is known from postoperative pain management, that when it occurs, it can disturb the patient's everyday life and reduce ability to work, and it is hard to treat [4, 15, 16].

We hypothesize that there are periprocedural predictors which can reveal the transition from acute to chronic pain state after PCI. Previous studies analysed the pain development only up to 2 days after PCI; therefore, we wanted to investigate if this pain persists for a longer period reaching the chronic stage at 3 months. Specifically, we aimed to assess the prevalence, pain control, and prognostic factors for chronic A-S pain development after PCI via radial artery access.

## 2. Materials and Methods

### 2.1. Study and Sample

This is a prospective observational study. The data of patients (*n* = 161), who underwent elective (nonemergency) PCI procedure using TR access at the Department of Cardiology (Hospital of Lithuanian University of Health Sciences), were collected in 2017 and analysed in 2020.

All patients were informed properly before the procedure and gave their written consent to participate in the study. The protocol was approved by the local institutional bioethics committee (protocol number BEC-MF-328).

**Figure 1:**
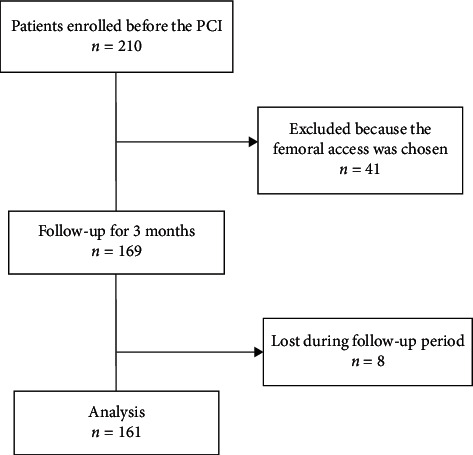
Study flowchart. PCI: percutaneous cardiac intervention.

Patients who underwent a PCI procedure and finished the follow-up period were consecutively included. Some patients were lost in the follow-up period because we were unable to contact them (wrong telephone number was given). The study flowchart is shown in Figure 1.

To determine prediction for A-S pain chronicity after PCI, the following factors were analysed: (1) demographic (patient's age, gender, body mass index >25, fear before the procedure question with possible answers Yes or No), (2) comorbid conditions (arterial hypertension, diabetes, dyslipidemia, depression, rheumatoid arthritis, carpal tunnel syndrome, and other comorbidities), and (3) periprocedural (pain intensity, intervention duration, arterial bleeding from puncture site, hand swelling following haemostasis, intervention wound pressure time after PCI, hematoma in puncture site, pseudoaneurysm, arterial thrombosis, arteriovenous fistula, neuropathy of the A-S area (hand), intervention site infection, and acute A-S pain after the procedure). Patients with limitation of a self-expression, severe psychiatric illness or <18 years old, and acute coronary syndrome (unstable angina and ST or non-ST elevated myocardial infarction) or III-IV NYHA class patients were excluded.

### 2.2. Patients and Public Involvement

Patients were involved in this study, and all of them gave written informed consent.

### 2.3. Measurements

Pain intensity was evaluated according to the Verbal Rating Scale (VRS): no pain—0, mild—1, moderate—2, severe—3, very severe—4, and worst possible pain—5 points. The follow-up of the pain prevalence and intensity were measured immediately after the procedure, 2 h, 12 h, 24 h, and 48 h and 3 months after PCI in hospital settings or interviewed throughout by telephone. Chronic A-S pain is described as a pain that lasts more than 3 months after PCI.

All patients were informed properly about the procedure before the PCI. An access method, a sheet size (6 F was used for all patients), shape of the guiding catheter, medical therapy, and other materials have been left to the discretion of the operator. The first step before puncture was to find the needed artery by anatomical orientations. Before sheet insertion, a local anaesthetic was injected underneath the skin (lidocaine solution 0.5–1 ml 1%). The operator has used the Seldinger technique for catheter insertion into the radial artery [17, 18]. PCI was performed by experienced interventional cardiologist. After PCI, haemostasis was provided by applying a pressure bandage (5 cm width, circular wrap) on the wrist at the puncture. It should be noted that the bandage started to loosen after 4 hours after the procedure and was continuously released until it was safe to remove completely. Hematoma is defined as the presence of a palpable mass greater than 3 cm in diameter measured by a measuring tape [19]. All complications were identified from the clinical picture and confirmed more detailedly by instrumental test methods (e.g., ultrasound) and by a specialist (neuropathy by neurologist, pseudoaneurysm by vascular specialist, etc.).

### 2.4. Data Analysis

The data analysis was performed with SPSS statistical software (v. 20.0 IBM). The sample size was calculated according to the prevalence of A-S pain after PCI found in the studies [9–14]. The confidence level of 95%, *α* is 0.05. Normally distributed continuous variables were presented as mean ± SD and compared using Student's *t*-test. Categorical data was presented as frequency and percentage and were statistically tested using Chi-square or Fisher's and Mann–Whitney test where it was appropriate. The univariate logistic regression analysis was used to identify prognostic factors for A-S pain and prognostic models. The relationship between the variables was assessed by the Spearman coefficient analysis. The correlation between the variables were ranked as weak for absolute values between 0 and 0.29, moderate—between 0.3 and 0.69, and strong—between 0.7 and 1.0. Missing data points were not imputed. All differences were considered statistically significant at *p* < 0.05.

## 3. Results

### 3.1. Study Sample

Clinical and procedural characteristics of patients (*n* = 161) are shown in Table 1. All patients had a history of IHD with a mean of 10 years.

### 3.2. Pain Development and Chronic Pain Predictors

**Figure 2:**
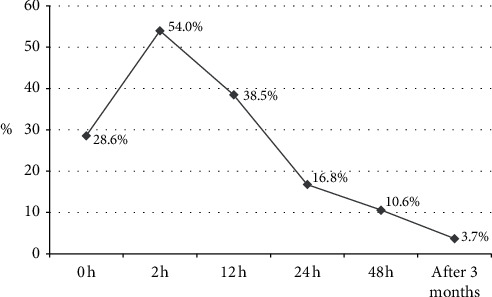
Development from acute to chronic stage after PCI: prevalence of access-site pain during 3-month follow-up.

The pain transition from acute to chronic is shown in Figure 2. The pain prevalence straight after the procedure was 29%, then jumped up to 54% in 2 hours, and decreased to 3.7% (6 patients) at the 3 months line after PCI. These 6 patients also reported persistent pain during the first 2 days after intervention, except for two patients who reported no pain at 48 hours after PCI, but at 3 months all 6 patients had A-S pain.

To see the potential predictors for acute pain following PCI, we analysed demographic and procedure-related factors. It was found that for acute pain the main consistent predictors were A-S complications after PCI, hematoma, and overall number of predictors (Table 2). These factors were related with 2 to 8 times higher odds for acute pain during 2–48 hours after PCI (*p* < 0.05). In addition to that, at very early stages after PCI (2–12 hours), the arterial bleeding from A-S and hand swelling following haemostasis were also strongly associated with the presence of acute pain. Note that the pressure bandage at the puncture site was kept tight for 6.74 (±1.77) hours after intervention. Also note that the fear before PCI procedure also had some predictive value for acute pain, but only to 12 hours after the procedure.

In order to see if the presence of pain across different follow-up points has predictive potential for pain persistence in the future, we calculated correlation matrix (Table 3). The results show that during the whole period under study, pain intensity correlations across different time points were medium (0.32 and higher). However, there was a trend that adjacent time point correlations were stronger (at levels 0.6-0.7). The chronic pain most strongly correlated with the pain intensity at 24 hours after procedure (which is = 0.47).

In this article, we also analysed the potential predictors for A-S pain chronicity. Here, we included such factors as demographic profile, comorbidities, procedure-related indicators, and acute pain characteristics. The findings revealed that the strongest and most consistent predictor of chronic pain development was acute pain after PCI procedure—starting from pain immediately after procedure to 48 hours following it (Table 4). The presence of acute pain predicted pain chronicity more strongly if measured at 24 and 48 hours after procedure (odds ratios 48.7 and 23.5, resp.), though earlier acute pain experienced after procedure was also significantly related to chronic pain development after 3 months (*p* < 0.05). The acute pain intensity was also a consistent predictor of the development of pain chronicity, increasing the odds by a factor of 2.56–3.58 (*p* < 0.05) per one additional intensity point increase (range from 0 to 5).

In addition to pain-related indicators, there were another three factors, significantly predicting the pain chronicity. These were A-S hand neuropathy (OR = 19.93), hematoma (OR = 6.48), and diabetes (OR = 5.77). It should be noted that A-S complications after PCI reached borderline significance for chronic pain with OR = 5.66 (*p* = 0.057). All other analysed indicators were not significantly associated with the development of pain chronicity after 3 months following PCI (Table 4).

### 3.3. Pain Control

The pain medication was prescribed by the attending physician when the patient complained about A-S pain. The prescription in the drug chart was noted “as needed.” The summary of the pain intensity and pain management for the 3-month period is demonstrated in Table 5. At different time points, pain treatment was prescribed to 9–24% of all patients undergoing PCI. In most cases, the NSAIDs were used (Ketoprofen and Ibuprofen); several cases received strong opioids (10 mg of morphine intravenously). For the majority of the cases, the patients experienced pain relief—from 50 to 100% of all pain-treated patients (Table 5).

## 4. Discussion

Radial access for the PCI highly reduces vascular complications compared with femoral access. There are approximately 2500 elective PCI via radial access performed per year at the Lithuanian University of Health Sciences Kaunas Clinics. The A-S pain after PCI can be caused by vascular complications (ischemia, thrombosis, and spasm), direct or indirect nerve damage, and postprocedural site care (haemostasis).

Zwaan et al. described upper extremity disfunction after PCI with prevalence up to 9.6% and showed that strong acute pain is one of the symptoms [9]. In the study from Cheng et al., postprocedural pain was evaluated after 3 and 24 hours. They found that pain intensity after 3 hours was 0–71 (range 0–100) and the median was 9. After 24 hours, pain intensity was decreasing to 0–40 (range 0–100), but more than 50% of patients were in mild–moderate pain [20]. Dharma et al. in the retrospective study concluded that the prevalence of strong A-S pain is 4.5%. The pain was evaluated 1 day after the procedure and the authors highlighted that initial data collection was not set to collect all the data that might interact with forearm pain [8]. The follow-up in our study was more than 3 months. We found that acute pain prevalence during the 48-hour time period was changing. The highest point was during 2 hours and after that point it was decreasing, but the intensity remained moderate throughout the whole follow-up period. Acute pain can be described as a cause of any injury, surgery, illness, trauma, or medical procedures, which lasts for a short period of time and disappears when the underlying cause is healed. It is known that acute pain can cause a stress-related physical response which can affect cardiopulmonary, metabolic, and other systems [21]. Different studies report different risk factors for acute A-S pain development after PCI. Those risk factors are low BMI, small wrist, gender (female), haemostasis, radial artery occlusion, hematoma, number of access attempts, and radial artery diameter [8, 22]. In our study, the main predictive factors for acute A-S pain were fear before the PCI, hand swelling following haemostasis, and the A-S complications after procedure (arterial bleeding and hematoma). It is notable that hematoma was a leading predictor throughout the follow-up period. Mechanism of arm swelling following haemostasis can be associated with the compression bandage. When it compresses too much, the return of venous blood is compromised. Swelling remained for 6 to 24 hours after the bandage was removed.

Pain management for IHD patients is very important. Not all patients from our study had pain relief medications. It can be associated with doctors' beliefs about pain after this procedure and cultural issues. Most Lithuanians tend not to disclose anxiety, depression, or pain. There is a common thought among patients that after a procedure or surgery, pain is to be expected, so in most cases, patients will only disclose that they are experiencing pain if that pain is unbearable. Our results show that most medications were NSAIDs, and strong opioids were used in several cases. NSAIDs have a larger negative impact on the cardiovascular system and incorrect use of opioids can lead to addiction. From the literature and clinical practice, it is known that the best pain management strategy is the multimodal approach, which can lead to better patient care after a procedure with prevention of chronic postprocedural pain development [5, 23].

Pain lasting longer than the normal healing period after surgery or procedure is an unwanted adverse event. The International Classification of Diseases Eleventh Revision (ICD-11) defines chronic postsurgical pain (CPSP) as pain which has developed after a surgical procedure and persisting beyond the healing process at least 3 months after surgery [24]. Today, most surgeries are modifying from open to minimally invasive procedures with expectance that patients will have less chronic pain and greater outcomes afterwards. In the literature, it can be found that even after those minimally invasive procedures, chronic pain can occur [25–28]. Even a minimally invasive procedure such as PCI, which is mostly considered nonpainful, requires more medical attention and multimodal analgesia is needed to prevent acute pain development. The multimodal pain management approach should be considered to reduce acute postprocedural pain prevalence. We found that A-S pain chronicity development after PCI is 3.7%. Predictive factors for the A-S pain chronicity development were diabetes, hand neuropathy, hematoma, and persistent pain after 12, 24, and 48 hours ad pain intensity during 48 hours after PCI.

The work from van De Kerkhof et al. states that the preoperative medical comorbidities and disability are risk factors to developing CPSP [29]. Diabetes in our observation was the predictive factor for the development of A-S pain chronicity after PCI. Krein et al. note that the growing number of people with diabetes and potentially particularly bad outcomes is without proper disease control and self-management. The authors note that the concomitant chronic pain also worsens the course of diabetes, reduces activity, and impairs self-care [30]. The effort to prevent the onset of chronic pain after PCI would contribute to proper diabetes management and vice versa.

Other very important predictive factors that we found for A-S pain chronicity were persistent pain during the 12–48 hours after PCI and pain intensity after 2–48 hours. Therefore, we found correlation between pain intensity and follow-up time points—2, 12, 24, and 48 hours and 3 months after PCI. The studies from postsurgical pain confirm that the main risk factor that will highly predict development of chronic pain after surgery is the severity of acute postoperative pain during the first days after surgery [31–33]. However, in each population, the predictive factors may vary; despite the fact that we used different haemostasis techniques and could not evaluate the exact number of access attempts, we were faced with familiar predictive factors in the acute pain stage. Our data indicates that the development of pain chronicity can be predicted based on periprocedural events: persistent pain in the acute period and the presence of complications and leading diseases—diabetes.

Neuropathic pain component in postoperative pain settings is the major factor to experiencing a higher pain intensity after operation, which can lead to chronic pain development after surgery [34]. In our study, hand neuropathy was diagnosed during the acute period and it is the predictor for A-S pain chronicity development after PCI. The mechanisms can be prolonged compression at the puncture site of radial artery which may result in damage or injury of the radial sensory nerve, causing neuropathic pain or hand dysfunction [35]. Radial compression may result in blood flow reduction into the surrounding tissues such as muscles and the lactate released from the ischemic muscle may cause ischemic pain by acting sensory neurons that innervates muscles [36]. In this study, we did evaluate possible nerve damage during PCI made by the interventional cardiologist which could be a direct risk factor for pain development. Other nerve injury causes can be hematoma, which can irritate or compress surrounding nerves.

Limitations of the study are that it is an observational single centre with a small-sized sample and the specific compression devices, pain management drugs, and time were individually applied and were not regulated by researchers. Also, some predictive factors (neuropathy and diabetes) were not investigated in detail, but our findings are still very important and can lead to a search for better assessment and development of the postprocedural pain assessment and management strategy in patients with IHD. Also, in this study, Verbal Rating Scale for pain evaluation was used which is based on subjective scale; thus, methodology bias cannot be ruled out. The choice by physician for analgesia was Ketoprofen and Ibuprofen, and we could not influence the choice.

In conclusion, the prevalence of chronic A-S pain is 3.7%. Pain during acute period was changing and most predictive factors were associated with periprocedural A-S complications. Hematoma was the leading predictive factor throughout the follow-up period. Predictive factors for A-S pain chronicity development were diabetes, neuropathy, hematoma, and persistent pain and pain intensity during the 48 hours after PCI. More observational studies should be carried out for IHD patient population with leading comorbidities requiring highly complex pain management. According to these findings, we have set a randomized controlled trial to check the hypothesis that multimodal pain management will reduce pain prevalence and pain intensity in acute phase with the goal being to prevent chronic pain development.

## Figures and Tables

**Table 1:** Clinical and procedural indicators.

Variable	*n*, % or mean ± SD	
*Clinical characteristics*		
Gender, female	63	39.1%
Age (years)	66.2 ± 10.59	
Diabetes mellitus	27	16.8%
Dyslipidemia	92	57.1%
Arterial hypertension	127	78.9%
Tunnel carpal syndrome before procedure	5	3.1%
Mean of IHD (years)	10.63 ± 11.07	
Depression	2	1.2%
Rheumatoid arthritis	4	2.5%
Body mass index (kg)	28.62 ± 4.77	
Smoking	50	31.1%
Fear before PCI	35	21.7%
The puncture site pain before PCI	5	3.1%

*Procedure*		
Mean of the procedure duration (minutes)	35.02 ± 24.63	
Mean time of the bandage removal (hours)	6.74 ± 1.77	
First time performed PCI	96	59.6%
PCI procedure performed more than once	65	40.4%
Access-site complications	46	28.6%
Arterial bleeding	15	9.3%
Hematoma	43	26.7%
Neuropathy	3	1.9%
Infection	0	0%
Artery thrombosis	0	0%
Pseudoaneurysm	0	0%
Arteriovenous fistula	0	0%
Hand swelling following haemostasis	107	66.5%

*Note*. IHD: ischemic heart disease. PCI: percutaneous cardiac intervention.

**Table 2:** Predictors for acute access-site pain following percutaneous coronary intervention.

Predictors	Pain development											
	After 2 h			After 12 h			After 24 h			After 48 h		
	OR	95% CI	*p*	OR	95% CI	*p*	OR	95% CI	*p*	OR	95% CI	*p*
Age	1.00	0.98–1.03	0.601	1.00	0.97–1.04	0.553	0.98	0.95–1.02	0.560	0.97	0.93–1.02	0.335
Gender (female)	2.50	1.23–5.08	0.011	1.67	0.83–3.36	0.147	2.33	0.93–5.83	0.070	2.64	0.86–8.08	0.088
Fear before PCI	4.53	1.84–11.14	0.001	3.63	1.66–7.93	0.001	1.33	0.51–3.47	0.556	0.75	0.20–2.79	0.673
Procedure duration	0.99	0.98–1.01	0.697	1.00	0.98–1.01	0.895	1.01	0.99–1.02	0.169	1.00	0.98–1.02	0.717
Times that performed PCI	0.79	0.42–1.50	0.485	1.23	0.64–2.35	0.525	0.85	0.361–1.99	0.709	0.79	0.27–2.27	0.667
Bandage removal duration	0.98	0.93–1.034	0.500	1.00	0.95–1.05	0.977	1.01	0.94–1.08	0.737	0.99	0.92–1.08	0.979
Access-site complications after PCI	6.68	2.80–15.89	<0.001	4.34	2.06–9.13	<0.001	6.18	2.44–15.66	<0.001	7.74	2.45–24.43	<0.001
Arterial bleeding from access-site	14.20	1.79–112.23	0.012	3.56	1.13–11.23	0.030	1.41	0.35–5.58	0.625	0.60	0.17–3.10	0.545
Hematoma	3.40	1.53–7.53	0.003	2.67	1.27–5.58	0.009	6.05	2.35–15.57	<0.001	6.85	2.17–21.60	0.001
Hand swelling following haemostasis	3.19	1.60–6.32	0.001	3.53	1.64–7.58	0.001	1.99	0.75–5.30	0.165	1.80	0.55–5.84	0.328

*Note*. IHD: ischemic heart disease. PCI: percutaneous cardiac intervention.

**Table 3:** Associations of access-site pain intensity after PCI during the follow-up: Spearman's correlation.

Time after PCI	0 h	2 h	12 h	24 h	48 h	3 months
0 h	—	0.47	0.37	0.37	0.40	0.30
2 h	0.47	—	0.70	0.49	0.36	0.26
12 h	0.37	0.70	—	0.58	0.43	0.32
24 h	0.37	0.49	0.58	—	0.71	0.47
48 h	0.40	0.36	0.43	0.71	—	0.38
3 months	0.30	0.26	0.32	0.47	0.38	—

PCI: percutaneous coronary intervention, all *p* < 0.001.

**Table 4:** Prognostic factors for prediction of access-site pain chronicity development.

Prognostic factors	Value	OR	95% CI	*p*
Age	Years	1.00	0.93–1.09	0.825
Gender	Female	3.64	0.57–23.46	0.174
BMI	25–29.99	0.62	0.84–4.60	0.642
	30+	0.68	0.09–5.11	0.714
Smoking	Present	0.42	0.04–4.10	0.460
IHD	Present	0.60	0.34–1.07	0.085
Diabetes	Present	5.77	1.07–31.08	**0.041**
Hypertension	Present	1.34	0.15–11.9	0.791
Dyslipidemia	Present	0.74	0.15–3.82	0.725
Other comorbidities	Present	0.46	0.05–4.29	0.499
Fear before PCI	Present	1.83	0.32–10.49	0.493
Previous history of PCI	Yes	0.73	0.12–4.10	0.717
Procedure duration	Minutes	1.01	0.99–1.03	0.129
Bandage removal duration	Minutes	0.95	0.84–1.09	0.522
Access-site complications after PCI	Present	5.66	0.95–33.75	0.057
Arterial bleeding from access-site	Present	1.96	0.19–19.30	0.561
Hematoma	Present	6.48	1.06–39.65	**0.043**
Neuropathy	Present	19.93	1.27–312.32	**0.033**
Hand swelling following haemostasis	Present	0.99	0.18–5.64	0.991

Access-site pain				
Immediately after PCI	Present	14.60	1.63–130.27	**0.016**
After 2 h	Present	—	—	—
After 12 h	Present	17.21	1.59–185.27	**0.019**
After 24 h	Present	48.74	4.87–487.2	**0.001**
After 48 h	Present	23.46	3.81–144.1	**0.001**

Pain intensity				
Immediately after PCI	VRS	3.30	1.65–6.60	**0.001**
After 2 h	VRS	2.56	1.15–5.73	**0.022**
After 12 h	VRS	3.02	1.70–5.39	**<0.001**
After 24 h	VRS	3.58	1.90–6.74	**<0.001**
After 48 h	VRS	2.89	1.72–4.87	**<0.001**

BMI—body mass index; IHD— ischemic heart disease; PCI— percutaneous cardiac intervention; and VRS— Verbal Rating Scale (0—no pain, 1—mild, 2—moderate, 3—strong, 4—very strong, and 5—worst possible pain).

**Table 5:** Pain intensity and its control after PCI.

Time after PCI	Pain intensity (VRS), median (IQR)	Pain treatment, *n* (%)	Medication used	Pain relief >50%, *n* (%)
0 h	2 (2–3)	8 (17.4)	NSAIDs	5 (62.5)
2 h	2 (1–2)	8 (9.2)	NSAIDs	5 (62.5)
12 h	2 (1–3)	7 (11.3)	NSAIDs	6 (85.7)
24 h	2 (1–3)	4 (14.8)	NSAIDs, strong opioids	2 (50.0)
48 h	2 (1–4)	4 (23.5)	NSAIDs	3 (75.0)
After 3 months	1.5 (1–3)	1 (16.7)	NSAIDs	1 (100.0)

*Note*. PCI: percutaneous coronary intervention; VRS: Verbal Rating Scale (1—mild, 2—moderate, 3—strong, 4—very strong, and 5—worst possible pain); IQR: interquartile range; and NSAID: nonsteroid anti-inflammatory medication.

## Data Availability

In order to guarantee the confidentiality and anonymity of the participants, data cannot be made publicly available. For more information, the corresponding author can be contacted.

## References

[1] Cho E. J., Yang J. H., Song Y. B. (2013). Type II complex regional pain syndrome of the hand resulting from repeated arterial punctures during transradial coronary intervention. *Catheterization and Cardiovascular Interventions: Official Journal of the Society for Cardiac Angiography & Interventions*.

[2] Bazemore E., Mann J. T. (2005). Problems and complications of the transradial approach for coronary interventions: a review. *The Journal of Invasive Cardiology*.

[3] Shroff A., Siddiqui S., Burg A., Singla I. (2013). Identification and managment of complications of transradial procedures. *Current Cardiology Reports*.

[4] Kanei Y., Kwan T., Nakra N. C. (2011). Transradial cardiac catheterization: a review of access site complications. *Catheterization and Cardiovascular Interventions*.

[5] Michelle M. L., Turner H. N., Collins P. M., Doellman D., Wrona S., Reynolds J. (2011). Procedural pain management: a position statement with clinical practice recommendations. *Pain Management Nursing*.

[6] Papadimos T. J., Hofmann J. P. (2002). Radial artery thrombosis, palmar arch systolic blood velocities, and chronic regional pain syndrome 1 following transradial cardiac catheterization. *Catheterization and Cardiovascular Interventions*.

[7] Sasano N., Tsuda T., Sasano H., Ito S., Sobue K., Katsuya H. (2004). A case of complex regional pain syndrome type II after transradial coronary intervention. *Journal of Anesthesia*.

[8] Dharma S., Kedev S., Patel T., Gilchrist I. C., Rao S. V. (2019). The predictors of post-procedural arm pain after transradial approach in 1706 patients underwent transradial catheterization. *Cardiovascular Revascularization Medicine*.

[9] Zwaan E. M., Koopman A. G. M. M., Holtzer C. A. J. (2015). Revealing the impact of local access-site complications and upper extremity dysfunction post transradial percutaneous coronary procedures. *Netherlands Heart Journal*.

[10] Benit E., Missault L., Eeman T. (1997). Brachial, radial, or femoral approach for elective Palmaz-Schatz stent implantation: a randomized comparison. *Catheterization and Cardiovascular Diagnosis*.

[11] Tharmaratnam D., Webber S., Owens P. (2010). Adverse local reactions to the use of hydrophilic sheaths for radial artery canulation. *International Journal of Cardiology*.

[12] Hildick-Smith D. J. R., Walsh J. T., Lowe M. D., Shapiro L. M., Petch M. C. (2004). Transradial coronary angiography in patients with contraindications to the femoral approach: an analysis of 500 cases. *Catheterization and Cardiovascular Interventions*.

[13] Ahmed W. H. (2003). Transradial coronary angiography and intervention. *Saudi Medical Journal*.

[14] Sciahbasi A., Fischetti D., Picciolo A. (2009). Transradial access compared with femoral puncture closure devices in percutaneous coronary procedures. *International Journal of Cardiology*.

[15] Jang H.-J., Kim J.-Y., Han J. D. (2016). Numbness after transradial cardiac catheterization: the results from a nerve conduction study of the superficial radial nerve. *Korean Circulation Journal*.

[16] SanmartínLee M., Cuevas D., Goicolea J., Ruiz-Salmerón R., Gómez M., Argibay V. (2004). Complicaciones vasculares asociadas al acceso transradial para el cateterismo cardíaco. *Revista Española de Cardiología*.

[17] Seldinger S. I. (1953). Catheter replacement of the needle in percutaneous arteriography: a new technique. *Acta Radiologica*.

[18] Pancholy S. B., Sanghvi K. A., Patel T. M. (2012). Radial artery access technique evaluation trial: randomized comparison of seldinger versus modified seldinger technique for arterial access for transradial catheterization. *Catheterization and Cardiovascular Interventions*.

[19] Pancholy S., Coppola J., Patel T., Roke-Thomas M. (2008). Prevention of radial artery occlusion-patent hemostasis evaluation trial (PROPHET study): a randomized comparison of traditional versus patency documented hemostasis after transradial catheterization. *Catheterization and Cardiovascular Interventions*.

[20] Cheng K. Y., Chair S. Y., Choi K. C. (2013). Access site complications and puncture site pain following transradial coronary procedures: a correlational study. *International Journal of Nursing Studies*.

[21] Gatchel R. J., Reuben D. B., Dagenais S. (2018). Research agenda for the prevention of pain and its impact: report of the work group on the prevention of acute and chronic pain of the federal pain research strategy. *The Journal of Pain*.

[22] Ferrante G., Rao S. V., Jüni P. (2016). Radial versus femoral access for coronary interventions across the entire spectrum of patients with coronary artery disease. *JACC: Cardiovascular Interventions*.

[23] Anderson K. O., Green C. R., Payne R. (2009). Racial and ethnic disparities in pain: causes and consequences of unequal care. *The Journal of Pain*.

[24] Treede R. D., Rief W., Barke A. (2015). A classification of chronic pain for ICD-11. *Pain*.

[25] McCormack K., Scott N. W., Go P. M., Ross S., Grant A. M. (2003). Laparoscopic techniques versus open techniques for inguinal hernia repair. *The Cochrane Database of Systematic Reviews*.

[26] Karthikesalingam A., Markar S. R., Holt P. J., Praseedom R. K. (2010). Meta-analysis of randomized controlled trials comparing laparoscopic with open mesh repair of recurrent inguinal hernia. *British Journal of Surgery*.

[27] Bignell M., Partridge G., Mahon D., Rhodes M. (2012). Prospective randomized trial of laparoscopic (transabdominal preperitoneal-TAPP) versus open (mesh) repair for bilateral and recurrent inguinal hernia: incidence of chronic groin pain and impact on quality of life: results of 10 year follow-up. *Hernia*.

[28] Bruce J., Quinlan J. (2011). Chronic post surgical pain. *Reviews in Pain*.

[29] VanDenKerkhof E. G., Peters M. L., Bruce J. (2013). Chronic pain after surgery. *The Clinical Journal of Pain*.

[30] Krein S. L., Heisler M., Piette J. D., Makki F., Kerr E. A. (2005). The effect of chronic pain on diabetes patients’ self-management. *Diabetes Care*.

[31] Althaus A., Hinrichs-Rocker A., Chapman R. (2012). Development of a risk index for the prediction of chronic post-surgical pain. *European Journal of Pain*.

[32] Gerbershagen H. J. (2013). Chronifizierung postoperativer Schmerzen. *Der Schmerz*.

[33] Katz J., Seltzer Z. E. (2009). Transition from acute to chronic postsurgical pain: risk factors and protective factors. *Expert Review of Neurotherapeutics*.

[34] Lavand’homme P. M., Grosu I., France M. N., Thienpont E. (2014). Pain trajectories identify patients at risk of persistent pain after knee arthroplasty: an observational study. *Clinical orthopaedics and related research*.

[35] Lubahn J. D., Cermak M. B. (1998). Uncommon nerve compression syndromes of the upper extremity. *Journal of the American Academy of Orthopaedic Surgeons*.

[36] Immke D. C., McCleskey E. W. (2001). Lactate enhances the acid-sensing Na^+^ channel on ischemia-sensing neurons. *Nature Neuroscience*.

